# The effects of field-based repeatedsprint training on physical performance in soccer players: a systematic review and multilevel meta-analysis

**DOI:** 10.3389/fphys.2026.1745959

**Published:** 2026-03-30

**Authors:** Anqi Chen, Mingnan Zhuang, Zijing Huang, Liang Zhao, Leibo Wang

**Affiliations:** 1 Graduate School of Shandong Sport University, Jinan, China; 2 Digital Physical Training Laboratory, Shandong Sport University, Rizhao, China; 3 School of Sport Training, Tianjin University of Sport, Tianjin, China; 4 School of Human Movement Science, Hebei Sport University, Shijiazhuang, China; 5 School of Athletic Performance, Shanghai University of Sport, Shanghai, China; 6 School of Competitive Sports, Shandong Sport University, Rizhao, China; 7 National Football Academy, Shandong Sport University, Rizhao, China

**Keywords:** athletic performance, repeated sprint ability, repeated sprint training, soccer, team sport

## Abstract

**Background:**

Repeated sprint training (RST) on the field has been employed as a time-efficient training strategy to enhance key physical performance in soccer players, such as short-sprint ability and repeated sprint ability (RSA). However, existing evidence regarding the effects of RST on comprehensive physical performance remains inconsistent, with previous reviews limited by methodological heterogeneity and insufficient statistical power. Therefore, this study aims to systematically review and quantify the effects of field-based RST on multiple physical performance indicators in soccer players through a multilevel meta-analysis.

**Methods:**

This study adhered to the PRISMA guidelines and was registered in PROSPERO. A systematic search was conducted across databases including PubMed, Web of Science, Scopus, and EBSCO from inception until August 2025. Randomized controlled trials comparing field-based RST with control training were included. Using the metafor package in R (version 4.2.1), a multilevel random-effects model was constructed to synthesize non-independent effect sizes and accurately estimate within-group correlations. Effect sizes were expressed as Hedges’ g with 95% confidence intervals. Parameters were estimated using restricted maximum likelihood, and the orchard package was employed for moderator analysis and result visualization. Heterogeneity was assessed via multilevel I^2^ statistics.

**Results:**

A total of 17 RCTs (386 participants) were included. The analysis revealed that RST significantly improved short-sprint ability (g = −0.26, 95% CI [–0.50, −0.03], p = 0.03), RSA (g = −0.37, 95% CI [–0.67, −0.08], p = 0.02), change of direction (CoD) ability (g = −0.70, 95% CI [–1.14, −0.25], p < 0.01) and high-intensity running (HIR) performance (g = 0.97, 95% CI [0.50, 1.43], p < 0.01). In contrast, RST did not yield significant overall effects on vertical jump (VJ) performance (g = −0.07, 95% CI [–0.40, 0.25], p = 0.64) or aerobic capacity (g = 0.02, 95% CI [–0.45, 0.50], p = 0.87). Most outcomes exhibited low heterogeneity (I^2^ ≤ 25.7%), with only RSA and VJ performance showing low-to-moderate heterogeneity.

**Conclusion:**

Field-based RST effectively enhances short-sprint ability, RSA, CoD ability and HIR performance in soccer players. However, its limited effects on VJ performance, and aerobic capacity. Future research should focus on clarifying dose–response relationships and integrating sport-specific movements and individual characteristics to optimize training prescriptions.

## Graphical Abstract

**Figure:**
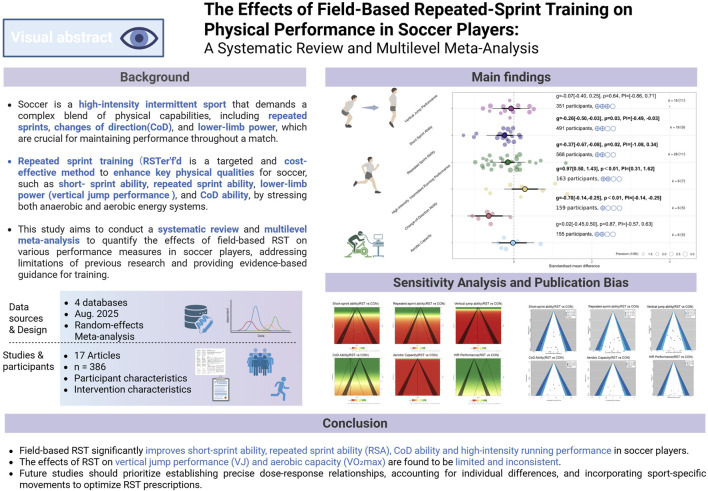

## Highlights

- Field-based repeated sprint training (RST) significantly enhances short-distance sprint performance, repeated sprint ability (RSA), change of direction (CoD) ability and high-intensity running (HIR) performance in soccer players, supporting its role as a sport-specific and time-efficient conditioning strategy.
- RST elicits only limited or inconsistent improvements in vertical jump performance, and aerobic capacity (VO_2_max), suggesting that its transfer to non-sprint-specific qualities may be constrained by movement pattern specificity and physiological demand.
- Future research should prioritize clarifying dose-response relationships, accounting for individual factors (e.g., age, sex, competitive level), and integrating sport-specific movements such as deceleration and directional changes to optimize training specificity and ecological validity.

## 1 Introduction

Soccer is a high-intensity intermittent sport which requires athletes to maintain the physical capacity to cope with dynamic changes throughout a 90-min match (Carling et al., 2008; Stølen et al., 2005). It is characterized by long periods of low-to moderate-intensity a interspersed with periods of high-intensity movements, (single and repeated sprints) (Clemente et al., 2021). Additionally, explosive actions, such as acceleration, deceleration, rapid directional changes, and jumpings, often occur in key moments of the match (Chtara et al., 2017). Repeatedsprint Ability (RSA)—the capacity to reproduce maximum or near-maximum speed efforts with short recovery—has been established as a key physical performance in elite soccer players (Rey et al., 2019). Moreover, essential technical actions during matches such as jumping, shooting, tackling, and quick turns, all rely on efficient explosive power output (Comfort et al., 2012; Manolopoulos et al., 2006). Therefore, systematically developing of strength, speed, power, and RSA is not only a core task of soccer conditioning but also the physiological foundation for maintaining high performance throughout a match.

High-Intensity Interval Training (HIIT), which includes repeatedsprint training (RST), sprint interval training, short-duration HIIT, long-duration HIIT, and small-sided games (SSGs), is an effective method for enhancing athletes’ strength, speed, power, and RSA (Buchheit and Laursen, 2013a; Thurlow et al., 2025b). Among these, SSGs and RST have become the most common and integral components of modern soccer conditioning programs due to their sport-specificity and time-/cost-efficiency (Buchheit et al., 2010; Bujalance-Moreno et al., 2019). Although SSGs are widely used to enhance technical-tactical skills and physical performance, systematic reviews suggest that achieving sufficient high-intensity running and sprinting stimuli often requires either a larger field or a reduction in the number of simultaneous participants—conditions that may be impractical for non-professional teams (Sarmento et al., 2018). The inherent variability in SSG design and the practical challenges in controlling their load highlight the need for complementary, standardized, and quantifiable high-intensity training methods.

In this context, RST—particularly field-based RST—has gained considerable attention due to its high ecological validity and compatibility with soccer training environments (Boer and Van Aswegen, 2016; Iaia et al., 2017). RST typically involves short all-out sprints (≤10 s) interspersed with brief recovery periods (≤60 s) (Bishop et al., 2011; Buchheit and Laursen, 2013b; Thurlow et al., 2023) and can be performed on the field, on a treadmill, on a cycle ergometer, or on a rowing ergometer (Taylor et al., 2015). Studies have shown that field-based RST can effectively improve short-distance sprint performance (Buchheit et al., 2010), RSA (Beato et al., 2022), lower-limb power (e.g., vertical jump performance), and change of direction (CoD) ability at the same time (Boer and Van Aswegen, 2016), it places considerable demand on both anaerobic and aerobic energy systems, promoting physiological adaptations such as enhanced buffering capacity and accelerated phosphocreatine resynthesis, making it a highly soccer-specific training strategy (Girard, et al., 2011; Mohr et al., 2005). Compared to treadmill-based protocols, field-based RST offers advantages in cost-effectiveness and applicability, meeting the needs of soccer players across different levels (Soares-Caldeira et al., 2014). Consequently, field-based RST has been systematically integrated into the conditioning programs of teams at various competitive levels. Through well-defined load structures and adjustable variables (e.g., sprint distance, rest intervals, training modality) (Bishop et al., 2011), field-based RST provides a precise means of delivering sufficient high-intensity running and sprinting stimuli, effectively compensating for the limitations of SSGs.

As the use of field-based RST has become increasingly common in professional soccer (Weldon et al., 2021; Iacono et al., 2023), its benefits for improving soccer players’ RSA and maximal sprint speed have been widely confirmed. However, the existing literature has not reached consistent conclusions regarding its effects on other physical performance indicators, such as maximal oxygen uptake (VO_2_max), vertical jump height (VJ height) (e.g., countermovement jump (CMJ) height, and agility. For example, Boer and Van Aswegen (2016) reported that RST significantly improved VO_2_max and VJ height in sub-elite soccer players, whereas Bravo et al. (2008) found that RST did not produce significant improvements in CMJ.

Existing meta-analyses have primarily focused on the effects of RST across different sports, often limited by substantial heterogeneity (I^2^ > 75%) and insufficient statistical power (Thurlow et al., 2024; Taylor et al., 2015). For instance, while Thurlow et al. (2024) noted positive effects of RST on VO_2_max and CMJ in athletes from various sports, high heterogeneity across studies left the magnitude of improvement for certain outcomes unclear. This suggests that previous meta-analyses—which often included athletes from multiple sports as the sample—may have limitations in data synthesis and effect-size estimation, making it difficult to accurately assess the overall efficacy of field-based RST for developing physical performance of athletes in a specific sport. Moreover, sport-specific meta-analyses focusing solely on soccer are relatively scarce, with existing reviews often integrating data across sports or focusing broadly on HIIT (Clemente et al., 2021).

Therefore, this study aims to conduct a systematic review and multilevel meta-analysis to quantify the effects of field-based RST on various physical performance indicators in soccer players, including short-distance sprint performance, RSA, vertical jump performance, CoD ability, aerobic capacity (VO_2_max), and high-intensity running performance (e.g., Yo-Yo Intermittent Recovery Test). By employing multilevel modeling to account for nested data structures—thereby addressing the heterogeneity issues present in prior research—this review will provide a more accurate and generalizable assessment of the effectiveness of RST and offer evidence-based guidance for integrating RST into soccer training programs.

## 2 Materials and methods

### 2.1 Protocol and registration

This systematic review was conducted in accordance with the Preferred Reporting Items for Systematic Reviews and Meta-Analyses (PRISMA®) guidelines (Page et al., 2021). To enhance transparency and reproducibility, the study was registered in the International Prospective Register of Systematic Reviews (PROSPERO ID: CRD42024609609).

### 2.2 Literature search

A systematic literature search was conducted independently by the first and third authors across multiple electronic databases, including PubMed, Web of Science (Core Collection), Scopus, and EBSCO. The search strategy used Boolean operators (“AND” and “OR”) to combine relevant subject headings and free-text terms. The search string included the following key concepts: [(RST OR “repeat* sprint*” OR “intermittent sprint*” OR “multiple sprint*” OR “sprint interval training”) AND (“exercise” OR “ability” OR “training”) AND (“team sport” OR “soccer” OR “football”) AND (“physiological” OR “metabolic” OR “fatigue” OR “performance”) NOT (“treadmill” OR “cycling” OR “swimming”)]. The search period was from the inception of the respective databases to August 2025. In addition to the electronic database search, manual screening of reference lists from eligible articles and previous meta-analyses was performed to identify further relevant studies. Further details regarding the search methodology are provided in Supplementary Material S2.

### 2.3 Inclusion and exclusion criteria

Literature selection adhered to the PICOS framework (Participants, Intervention, Comparators, Outcomes, Study design) (Methley et al., 2014). The inclusion criteria were: (1) All participants were soccer or football players, ranging from adolescents to adults across multiple competitive levels, including professional, semi-professional, amateur, and elite players. (2) randomized controlled trials (RCTs) published in English in peer-reviewed journals; (3) RST interventions comprising repeated maximal sprints (≤10 s) with between-repetition recovery ≤60 s; (4) comparison of RST with a control group performing regular soccer training or an alternative non-sprint training regimen; and (5) reporting of at least one soccer-specific physical performance measure (e.g., CMJ height). Exclusion criteria included: (1) non-RCTs, reviews, commentaries, or observational studies; (2) RST protocols employing treadmills, cycling, inclined surfaces, or other non-sport-specific modalities; applied modified RST protocols (e.g., using treadmills, bicycles, or inclined surfaces); (3) interventions involving atypical physiological stressors such as hypoxia, heat stress, or ergogenic aids; and (4) studies with incomplete or non-extractable outcome data necessary for meta-analysis.

### 2.4 Data selection

To eliminate duplicates, all literature retrieved from the databases was imported into EndNote X9 for deduplication. Two reviewers (C.A.Q. and Z.M.N), both trained in systematic evidence-based medicine, independently screened the titles and abstracts of the identified studies against the predefined eligibility criteria. Any disagreements were resolved through discussion with a third reviewer (H.Z.J.). In the full-text assessment phase, the same inclusion and exclusion criteria were applied independently by the reviewers to ensure consistency. The entire selection process was conducted with rigor and transparency. Inter-rater agreement was assessed using SPSS (version 26.0; IBM Corp., 2022), with a Kappa value of 1.00, indicating perfect agreement between the reviewers (Narducci et al., 2011).

### 2.5 Data extraction and transformation

Following screening, two reviewers independently extracted data utilizing a standardized form developed in Microsoft Excel (version 16.97). The extracted data encompassed the following domains: (a) study characteristics (first author, year of publication); (b) participant characteristics (sample size, age, body mass index, and health status); (c) intervention parameters (exercise modality, intensity, frequency, session duration, and total intervention period); and (d) outcome data, including pre- and post-intervention means and standard deviations for key performance metrics. To ensure consistent interpretation in the subsequent meta-analysis, the direction of the effect for time-based outcomes (e.g., short-sprint performance over 10–30m, CoD ability such as the Illinois Agility Test) was standardized so that negative values represent performance improvements (i.e., shorter completion times). In contrast, for outcomes where higher values indicate better performance (e.g., vertical jump performance like CMJ and SJ, aerobic capacity such as VO_2_max, and high-intensity running performance like the Yo-Yo IRT1), positive values were retained to denote improvement.

In instances where data were absent from the published report, the corresponding author was contacted via email to solicit the missing information. For outcomes presented exclusively in graphical form, data were extracted using WebPlotDigitizer (version 4.1; https://automeris.io/WebPlotDigitizer). Studies from which complete data could not be acquired were subsequently excluded from the quantitative synthesis.

For the analysis of soccer players’ physical performance, pre-intervention (baseline) and post-intervention values were extracted. The mean difference (MD) in outcome was calculated for each study as the difference between the post-intervention mean (Mpost) and the pre-intervention mean (Mpre), expressed as:

$${\mathrm{MD}}_{\mathrm{diff}}=M_{\mathrm{post}}-M_{\mathrm{pre}}$$

In studies where data were reported solely as confidence intervals (CIs), the values were converted to standard deviations (SD) using established computational methods. Where N represents the sample size, CI_high_ the upper confidence interval limit, CI_low_ the lower confidence interval limit, and C a constant value dependent on the specific confidence level and t-distribution. The conversion was performed according to the following formula:

$$SD=\sqrt{N}\;\frac{{CI}_{high}-{CI}_{low}}{2t}$$

When standard error (SE) was reported, it was converted to standard deviation (SD) using the formula:

$$SD=\sqrt{N}\times SE$$

The SD of the change was determined using the following formula.

$$SD_{diff}=\sqrt{SD_{pre}^{2}+SD_{post}^{2}-2r\times SD_{pre}\times SD_{post}}$$

### 2.6 Risk of bias assessment and certainty of evidence

The risk of bias in the included studies was evaluated using the Cochrane Risk of Bias tool (RoB 2.0), in accordance with the guidelines provided by the Cochrane Collaboration (Higgins et al., 2011). This tool assesses five domains for potential bias: (1) the randomization process; (2) deviations from intended interventions; (3) missing outcome data; (4) measurement of the outcome; and (5) selection of the reported result. Each domain was judged as “low risk,” “some concerns,” or “high risk,” leading to an overall risk-of-bias assessment for each study. Two independent reviewers (C.A.Q. and Z.M.N.) conducted the evaluations, and any disagreements were resolved through consultation with a third reviewer.

### 2.7 GRADE evidence assessment

Two independent reviewers (Z.L. and W.L.B.) evaluated the quality of evidence using the Grading of Recommendations, Assessment, Development, and Evaluations (GRADE) framework (Guyatt, 2008). The certainty of evidence was assessed across five key domains: risk of bias, inconsistency, indirectness, imprecision, and publication bias. Evidence was then classified into one of four certainty levels: high, moderate, low, or very low. The specific criteria for downgrading evidence certainty were predefined as follows: (a) Risk of bias: If studies were rated as having “some concerns” of bias, the certainty was downgraded by one level; if rated “high risk,” it was downgraded by two levels. (b) Inconsistency: Downgrading by one level was applied for moderate heterogeneity (I^2^ > 25%), and by two levels for substantial heterogeneity (I^2^ > 75%). (c) Imprecision: The certainty was downgraded by one level if the confidence interval was wide and crossed a prespecified clinically important threshold, or if the optimal information size was not met. (d) Publication bias: A significant result on Egger’s test (p < 0.05) led to a one-level downgrade.

### 2.8 Statistical analysis

All statistical analyses were conducted in R version 4.4.3 using the “*meta*” and “*metafor*” packages (Viechtbauer, 2010). A multilevel random-effects meta-analysis was employed to address the statistical non-independence arising from multiple effect sizes within individual studies (e.g., different outcome measures or time points). Traditional two-level meta-analyses may violate the assumption of effect size independence, thereby overestimating precision due to the duplicated data structure (Van den Noortgate et al., 2013). To address this issue, this study adopted the three-level random-effects meta-analytic framework proposed by Assink and Wibbelink (2016). This model decomposes the total variance into three levels: Level 1 represents sampling variance, Level 2 represents within-study variance, and Level 3 represents between-study variance, thereby effectively handling dependencies among effect sizes and the hierarchical data structure (Cheung, 2019).

To further control for statistical dependence, this study incorporated cluster-robust variance estimation (CRVE) based on the variance–covariance matrix, along with small-sample corrections, to obtain unbiased standard errors in the presence of correlated outcomes (Pustejovsky and Tipton, 2022). Compared to averaging or discarding multiple effect sizes from the same study, retaining all available effect sizes enhances statistical power and improves estimation accuracy (Assink and Wibbelink, 2016). Meanwhile, a random-effects model was applied to account for expected heterogeneity arising from differences in study design, intervention protocols, and participant characteristics. Model parameters were estimated using restricted maximum likelihood (REML), and cross-validation was performed via maximum likelihood (ML) to ensure robustness (Borenstein et al., 2010).

The effect size measure used was the standardized mean difference (SMD). Considering the small sample sizes in some included studies, Hedges' g was adopted as a bias-corrected standardized effect size and interpreted according to the following criteria: negligible effect (g < 0.2), small effect (0.2 ≤ g < 0.5), moderate effect (0.5 ≤ g < 0.8), and large effect (g ≥ 0.8) (Nagashima et al., 2019). Between-study heterogeneity was assessed using Cochrane’s Q-test, I^2^ statistic, τ^2^, and τ values, while the 95% prediction interval (PI) was also reported to reflect the distribution range of true effects (Nakagawa et al., 2017). In line with prevailing methodological recommendations, I^2^ served as the primary indicator of heterogeneity, with interpretation thresholds defined as follows: 0%–25% (low heterogeneity), >25–50% (moderate heterogeneity), >50–75% (substantial heterogeneity), and >75–100% (high heterogeneity) (Cumpston et al., 2019). Additionally, to evaluate the statistical power of the pooled effect and reduce the risk of type II error, a statistical power analysis was performed using the “metameta” package (Quintana, 2023).

The visualization is performed using a bubble plot (via the “orchard” package), where the size of the bubbles is proportional to the reciprocal of the standard error (1/SE). Publication bias was evaluated using enhanced funnel plots and Egger’s regression test (Wagner et al., 2018). When the multilevel data structure precluded the application of the standard Egger’s test, a linear model approximation was applied. If significant funnel plot asymmetry was detected, the trim-and-fill method was used to adjust the overall effect size estimate.

## 3 Results

### 3.1 Study selection

**FIGURE 1:**
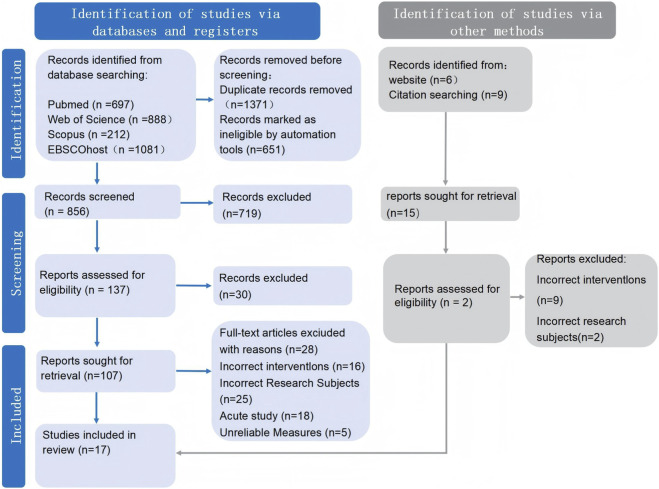
PRISMA flow diagram of study selection.

The initial search yielded 2,878 records. After removing duplicates using EndNote 856 articles remained for title and abstract screening. A total of 17 studies published between 2008 and 2025 were eligible for inclusion. All studies were published in English and collectively included 386 athletic participants. The study selection process is detailed in the PRISMA flow diagram (Figure 1). Inter-rater agreement for study selection was high, with a Kappa statistic of 0.870 (p < 0.01) initially, which reached perfect agreement (Kappa = 1.00, p < 0.01) after consultation with a third author (H.Z.J). The included studies were deemed methodologically sound, sufficiently homogeneous, and provided adequate data for the subsequent multilevel meta-analysis.

### 3.2 Study characteristics

This analysis included 17 randomized controlled trials, with a total of 386 participants (Table 1). The pooled sample had a mean (±SD) age, body mass, and height of 17.58 ± 3.04 years, 67.24 ± 9.70 kg, 168.18 ± 7.46 cm. Intervention durations ranged from 4 to 12 weeks, with typical training frequencies of 2–3 sessions per week. All RST interventions were field-based running. A standard RST session generally comprised 2 to 6 sets of 4–10 repetitions. Sprint distances were 10–40 m per repetition, with intra-set rest intervals of 20–30 s and inter-set recovery periods of 1–5 min. Primary outcomes included short-sprint performance (e.g., 30-m sprint time), RSA (assessed via best sprint time [RSAbest] and mean sprint time [RSAmean]), and vertical jump performance (e.g., countermovement jump [CMJ] and squat jump [SJ]). Aerobic capacity was evaluated using either direct measurements of VO_2_max (from expired gas analysis during laboratory-based incremental tests) or estimated values (e.g., equations applied to field-based tests). High-intensity intermittent running performance was also assessed using measures such as the Yo-Yo IR1/IR2 (Yo-Yo Intermittent Recovery Test Level 1 or 2) or 30-15IFT (30-15 Intermittent Fitness Test).

**TABLE 1:** Summary of study characteristics.

Author, Year	Group(n)	Subjects	Duration (weeks)	Frequency (times)	Repeated Sprint Training Paramaters	Outcome Measures
Ben Abderrahman et al. (2024)	RST (15) CON(15)	Tunisian soccer team, male, 21.8 ± 2.6years, 67.5 ± 7.4 kg, 179.6 ± 7.8 cm	8	3	2sets × 5reps × 20m, 15s rest, 1 min rest between sets	RSAbest, RSAdec, SJ, CMJ, 30 m
Boer and Van Aswegen (2016)	RST (17) CON(13)	Sub-elite male players 21.7 ± 1.8 years, 65.5 ± 8.1 kg, 173 ± 0.1 cm	6	3	3sets × 6reps × 40m, 10s rest, 4 min rest between sets	Yo-Yo IR2 test, VJ, 30m, T-test, VO_2_max
Bravo et al. (2008)	RST (13) CON(13)	Junior professional football players, 17.3 ± 0.6 years, 71 ± 5.6 kg, 179.3 ± 4.8 cm, the amateur players, 24.3 ± 5.4 years, 76.5 ± 5.4 kg, 179.4 ± 4.8 cm	7	2	3sets × 6reps × 40m, 20s rest, 4 min rest between sets	VO_2_max, CMJ SJ, 10 m Yo-Yo IR1 test, RSAmean
Buchheit et al. (2010)	RST (7) CON(8)	Twenty elite, male, adolescent players, 14.5 ± 0.5 years, 64 ± 8 kg, 1.74 ± 0.10	10	1	2-3sets × 5-6reps × 30–40m, 14s rest	10m, 30 m, RSAbest, RSAmean CMJ
Chtara et al. (2017)	RST (12) CON(10)	Elite soccer players, male, 13.6 ± 0.3-year, 1.65 ± 0.07 m, 54.1 ± 6.5 kg	6	2	2-4sets × 5-6reps × 20–30m, 20s rest, 1min rest between sets	10m, 30 m, ZIigzag 20m, RSAbest RSAmean
Eniseler et al. (2017)	RST (9) CON(10)	National level soccer players, 16.9 ± 1.1 years, 65.6 ± 5.6 kg, 174.3 ± 4.8 cm	6	2	3sets × 6reps × 40m, 20s rest, 4 min rest between sets	Yo-Yo IR1 TEST RSAdec, RSAmean RSAbest
Izquierdo et al. (2021)	RST (14) CON(13)	17.7 ± 0.7 years, 176.8 ± 6.3 cm, 69.9 ± 8.7 kg	6	2	2-4sets × 5-6reps × 20–30m, 20–30s rest, 4 min rest between sets	CMJ, RSAmean, RSAdec, RSAbest
Krakan et al. (2020)	RST (20) CON(21)	Amateur to semi-pro level, male RST group, 175.36 ± 6.19 cm, 77.29 ± 9.50 kg CON group, 181.23 ± 6.92 cm, 80.54 ± 8.12 kg	6	3	2-3sets × 6reps × 20 m	CMJ, 5m, 10m, 25 m MAG20y, RSAbest RSAmean, RSAdec VO2max
Nascimento et al. (2015)	RST (8) CON(6)	Amateur male players, 16.7 ± 0.5 years, 68.5 ± 6.6 kg, 176.6 ± 4.5 cm	4	2	3sets × 6reps × 40m, 20s rest, 4 min rest between sets	CMJ
Nedrehagen and Saeterbakken (2015)	RST (12) CON(10)	Amateur team playing at the local level, Male, 22.0 ± 2.7,180.7 ± 3.4,77.6 ± 8.9 female,19.9 ± 2.5,168.1 ± 6.0,61.5 ± 9.1	8	1	3-4sets × 4-6reps, 30m, 30s rest, 5min rest between sets	Yo-Yo IR1 TEST RSAmean
Nobari et al. (2023)	RST (14) CON(14)	RST group, 17.2 ± 0.4 years 179.6 ± 6.9 cm 69.6 ± 9.6 kg CON group, 17.2 ± 0.4years 176.5 ± 5.0 cm 63.1 ± 7.2 kg	4	2	3sets × 6reps × 40m, 10s rest, 4 min rest between sets	Sargent jump test, 10 m 20m, 30m, 505 cod test 30-15 IFT test
Perween et al. (2020)	RST (10) CON(10)	University level athletes, male, RST group, 20.7 ± 0.63 1.71 ± 0.02 64.98 ± 3.77 CON group, 19.70 ± 0.47 1.68 ± 0.01 61.29 ± 1.61)	4	3	3sets × 6reps × 40m, 10s rest, 4 min rest between sets	VO2max
Sanchez-Sanchez et al. (2019)	RST (10) RST (10) CON(9)	Regional-level, male, CON group, 14.9 ± 0.4 years; 169.1 ± 6.8 cm 62.5 ± 7.1 kg RST-HAF group, 14.7 ± 0.5 years; 169.7 ± 7.6 cm; 62.7 ± 8.8 kg RST-lAF group, 14.4 ± 0.5 years, 169.1 ± 5.8 cm, 58.4 ± 6.6 kg,	8	2	3sets × 10reps × 18m, 10 m rest, 4 min rest between sets	RSAbest, RSAmean RSAdec, VO2max Yo-Yo IR TEST COD test
Selmi et al. (2018)	RST (15) CON(15)	Elite soccer players, male, 17.8 ± 0.9 years; 1.78 ± 0.05 m; 70.1 ± 6.6 kg,	6	3	2-3sets × 5-6reps × 30–40m, 20s rest, 4 min rest between sets	RSAbest, RSAdec
Soares-caldeira et al. (2014)	RST (7) CON(7)	Professional level, male, 21.4 ± 5.5 years, 72.2 ± 8.8 kg, 172.3 ± 5.7 cm	4	2	2sets × 6-8reps × 30m, 20s rest, 5min rest between sets	Yo-Yo IR1 test, SJ CMJ, RSAbest RSAmean, RSAdec
Thurlow et al. (2025a)	RST (12) CON(12)	Elite level, male 20.8 ± 0.8 182 ± 5 90.4 ± 13.4	6	2	2-3sets × 6-8 reps × 30–40m, 30s rest, 2min rest between sets	CMJ, 10m, 20m, 30 m
Venturelli et al. (2008)	RST (7) CON(9)	Professional level, Male, 11.0 ± 0.5 years, 40.5 ± 5.0 kg, 149.0 ± 6.0 cm	12	2	20reps × 10–20m, 60–90s rest	20 m (with ball/without ball), SJ, CMJ

RST, repeatedsprint training group; CON, control group; M, male; F, female; CMJ, counter movement jump; SJ, squat jump; VO_2_max, maximum oxygen uptake; Yo-Yo IR1/IR2, Yo-Yo Intermittent Recovery Test Level 1 or 2; RSAbest, the best sprint time in the Repeated Sprint Test; RSAmean, the average sprint time in the Repeated Sprint Test; RSAdec, the rate of speed decrement in the Repeated Sprint Test.

### 3.3 Risk of bias assessment

**FIGURE 2:**
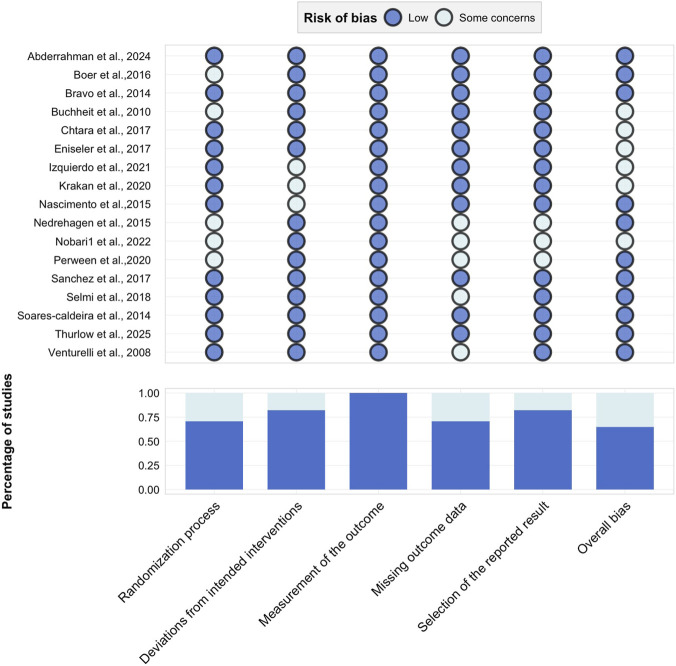
Risk of bias assessment graph for included studies.

The risk of bias assessment for the included studies is summarized in Figure 2. Detailed evaluation across five domains revealed the following: the randomization process was rated as low risk in 70.59% of studies, though five studies did not adequately describe random sequence generation, indicating potential selection bias. Deviations from intended interventions were assessed as low risk in 82.35% of studies, with 17.65% rated as having some concerns and none as high risk. Measurement of the outcome was judged as low risk across all studies (100%). For missing outcome data, 70.59% of studies were low risk, 29.41% had some concerns, and none were high risk. In the selection of the reported result, 82.35% were low risk, 17.65% had some concerns, and no studies were high risk. Overall, the majority of included studies were considered to have a low risk of bias.

### 3.4 Meta-analyses results

This meta-analysis aimed to quantify the overall effects of field-based RST on physical performance in soccer players. A multilevel random-effects model was employed to synthesize outcomes across studies, comparing RST interventions against control conditions, which generally consisted of regular soccer training or alternative non-sprint training protocols.

**FIGURE 3:**
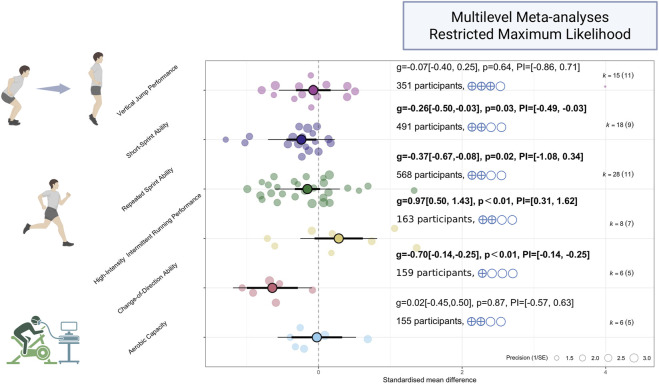
Forest plot summarizing the effects of RST on soccer players' Physical Performance. The forest plot displays the individual effect sizes (coloured dots) from each study and the pooled overall effect estimate (black diamond with 95% confidence interval) of RST on Physical Performance outcomes in soccer players. The size of each data point is proportional to the precision of the effect estimate (1/standard error). The vertical dashed line represents the line of null effect (Hedges' g = 0). For sprinting and CoD ability (time-based outcomes), effect sizes falling to the left of the zero line indicate an advantage for the RST intervention group (i.e., shorter times). For non-time-based outcomes such as vertical jump height and high-intensity running capacity, effect sizes falling to the right of the zero line indicate an advantage for the RST intervention group (i.e., superior performance).Translated with DeepL.com (free version) The annotations on the right, denoted as k = X (Y), indicate the number of included studies (X) and the corresponding number of effect size clusters (Y) for each outcome.

The results demonstrated that RST elicited statistically significant improvements in several performance metrics: short-sprint ability (k = 18, Hedges’ g = −0.26, 95%CI [−0.50, −0.03], PI [−0.50, −0.03, p = 0.03), RSA (k = 28, Hedges’ g = −0.37, 95%CI [−0.67, −0.08], PI [−1.08, 0.34], p = 0.02), CoD ability (k = 6, Hedges’ g = −0.70, 95%CI [−1.14, −0.25], PI [−1.14, −0.25], p = <0.01), and high-intensity running performance (k = 8, Hedges’ g = 0.97, 95%CI [0.50, 1.43], PI [0.31, 1.62], p < 0.01). In contrast, no significant overall effects were observed for vertical jump performance (k = 15, Hedges’ g = −0.07, 95%CI [−0.40, 0.25], PI [−0.86, 0.72], p = 0.64)or aerobic capacity (k = 6, Hedges’ g = 0.02, 95%CI [−0.45, 0.50], PI [−0.57, 0.63, p = 0.87). See in Figure 3.

No substantial heterogeneity was observed for short-sprint performance (I^2^ = 0%), high-intensity running performance (I^2^ = 25.7%), aerobic capacity (I^2^ = 0%), or agility (I^2^ = 0%). In contrast, low to moderate heterogeneity was detected for RSA (I^2^ = 45.4%) and lower-limb power (I^2^ = 44.7%). Sensitivity analysis using the “leave-one-out” method identified no outliers across any of the outcome measures.

**FIGURE 4:**
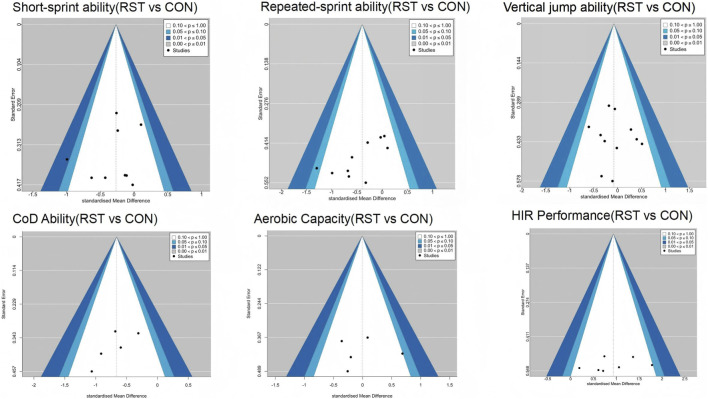
Summary Funnel Plot of RST Effects on soccer players' Physical Performance.

Publication bias, as assessed by Egger’s test, was significant only for RSA (p = 0.030). No substantial bias was indicated for the remaining outcomes, including short-sprint performance (p = 0.44), high-intensity running performance (p = 0.41), aerobic capacity (p = 0.97), lower-limb power (p = 0.802), and agility (p = 0.234). Effect sizes for the significant outcomes, based on standardized mean differences, ranged from small to large. See in Figure 4.

### 3.5 GRADE evidence evaluation results

The certainty of evidence for each outcome was assessed using the GRADE approach. Evidence for the effects on short-sprint ability, RSA, CoD performance, aerobic capacity, and HIR performance was rated as low. This rating was mainly attributable to substantial heterogeneity (inconsistency) observed for short-sprint ability and RSA outcomes, as well as imprecision due to wide confidence intervals for CoD performance, aerobic capacity, and HIR performance. Evidence for VJ performance was judged as moderate but was downgraded due to imprecision, as the confidence intervals encompassed the possibility of no effect. No other significant limitations were identified across the assessed domains. Further methodological details are available in Supplementary Material S3.

### 3.6 Sensitivity analysis and publication bias

After excluding individual studies one by one, the overall effect size and its significance did not undergo substantial changes, indicating that the results of this study are highly robust. In terms of publication bias testing, a funnel plot was used to visually examine the distribution of effect sizes across studies. The results showed that the distribution was generally symmetrical, with only minor deviations observed in some small-sample studies, as shown in Figure 4.

**FIGURE 5:**
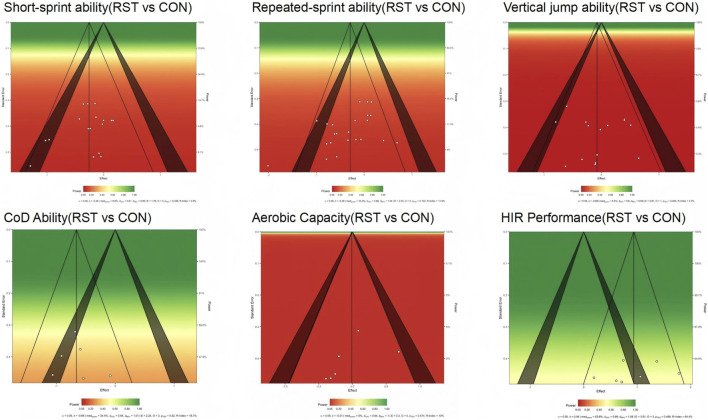
Sunset Plot of RST Effects on soccer players' Physical Performance. The sunset plot simultaneously displays the statistical power of included studies and potential publication bias. The color gradient indicates levels of statistical power, transitioning from red (low power) to green (high power). Individual studies are represented by white dots, positioned according to their effect sizes and standard errors.

Figure 5 displays the comprehensive main effect statistical power assessed through a sunset plot. Detailed forest plots for each outcome measure are provided in Supplementary Material S3.

## 4 Discussion

### 4.1 Effects of field-based RST on short- sprint ability

The results of the meta-analysis reveal that field-based RST leads to small improvements in short-sprint performance. Specifically, RST induces statistically significant physiological adaptations that effectively enhance sprint times over 10 m, 20 m, and 30 m, consistent with previous meta-analytical findings on the effects of RST on short-distance sprint ability (Taylor et al., 2015). During the process of short-distance sprints, achieving and maintaining maximum sprint speed is key to enhancing performance in a single sprint (Bishop et al., 2011). Previous studies have identified several key factors that influence maximal velocity, including the depletion of anaerobic metabolic substrates, muscle activation, and motor unit recruitment strategie (Girard et al., 2011). The improvement in sprinting performance following RST is accompanied by several physiological adaptations, including beneficial increases in muscle metabolites (such as phosphocreatine and glycogen) and enzyme activity (Rodas et al., 2000). These physiological adaptations resulting from RST may help explain the observed improvements in sprint performance. Furthermore, the observed improvements in sprinting ability may partly be attributed to the activation of relatively inactive or undertrained muscle groups by RST, leading to adaptive changes in muscle function as well as the enhancement of the contraction characteristics of the leg extensors, which helps generate greater force (Lieberman et al., 2006). Although the specific mechanisms underlying these adaptive changes are not yet fully understood, these findings have been emphasizing the importance of RST in enhancing anaerobic capacity in athletes, particularly in terms of short-distance sprinting and explosive power in soccer.

The present study did not observe significant improvements in 5 m sprint performance following RST (p > 0.05, 0.89%), although this conclusion is drawn from only one study that included a 5 m test (Krakan et al., 2020). Current evidence suggests that 5-m sprint performance primarily relies on the ability to generate horizontal acceleration (Majumdar and Robergs, 2011). Given the limited available evidence, it is premature to draw definitive conclusions regarding the efficacy of RST for 5 m sprint performance. Future research should employ more rigorously designed studies to systematically evaluate the effects of RST on sprint performance over this phase. A promising direction involves combining RST with strength and power training (e.g., resisted sprint training). Such combined training models may enhance short-distance acceleration by improving muscular mechanical output. Subsequent studies should focus on elucidating the specific mechanisms by which RST influences athletic performance during the initial acceleration phase.

### 4.2 Effects of field-based RST on RSA

The findings of this present study demonstrate that field-based RST is an effective intervention for enhancing RSA in soccer players. Multilevel analyses revealed statistically significant improvements in RSAbest, RSAmean, and RSAdec. The effectiveness of RST is primarily underpinned by the principle of task specificity, whereby the training stimulus closely emulates the biomechanical, neural, and energetic demands of sprinting encountered during actual match-play (Bishop et al., 2011). This alignment between training and performance context facilitates specific physiological adaptations that translate into improved RSA, mediated through the following mechanisms. RSA is dependent upon the integrated performance of metabolic and neuromuscular systems (Girard et al., 2011; Enoka and Stuart, 1992). Metabolically, RST elicits substantial adaptations: the cyclical nature of maximal sprints interspersed with brief recovery periods (<60s) enhances phosphocreatine resynthesis rates, improves muscle buffering capacity, and upregulates the activity of key anaerobic enzymes such as phosphorylase and phosphofructokinase (Matsuura et al., 2007; Gaitanos et al., 1991). These adaptations collectively promote more efficient energy regeneration and better maintenance of intracellular homeostasis during successive sprint efforts. From a neuromuscular standpoint, repeated maximal efforts optimize motor unit recruitment patterns—specifically enhancing the synchronization and recruitment of high-threshold motor units—and improve inter-segmental coordination as well as lower-limb stiffness during swing and ground contact phases (Casey et al., 1996; Wilson and Murphy, 1996). Moreover, the prescribed insufficient recovery intervals induce accumulated metabolic stress (e.g., from inorganic phosphate [Pᵢ] and hydrogen ion [H^+^] accumulation), challenging the neuromuscular system to preserve motor unit firing rates and contractile function under fatigued conditions (Spriet et al., 1989; Thomas et al., 2005). This process directly simulates the high-intensity intermittent endurance demands characteristic of soccer match-play. These concurrent adaptations in metabolic and neuromuscular systems collectively form the physiological foundation that enables the maintenance of peak sprint velocity and minimizes performance decrement across multiple sprint bouts.

### 4.3 Effects of field-based RST on vertical jump performance

This meta-analysis found that field-based RST did not produce statistically or practically significant improvements in VJ performance, with only a trivial effect size (g = 0.07). This finding diverges from previous meta-analytical conclusions (Taylor et al., 2015), and the underlying mechanisms can be explained from multiple biomechanical and physiological perspectives. Primarily, the specificity of neuromuscular demands appears to play a central role: VJ necessitates rapid triple extension with high-rate force development under brief ground contact conditions (Sierer et al., 2008), whereas RST predominantly emphasizes horizontal propulsion and longer ground contact periods, thereby failing to adequately replicate the distinct force-vector and temporal characteristics of jumping tasks. From a neuromuscular adaptation perspective, the limited stimulus for stretch-shortening cycle enhancement under high eccentric loads further explains this lack of transfer (Bishop et al., 2011). Most RST protocols focus on linear acceleration and maintenance of sprint velocity rather than incorporating rapid deceleration–reacceleration cycles or high-intensity rebound actions, which are essential for inducing adaptations in tendon elasticity and reactive strength (Markovic and Mikulic, 2010).

Additionally, the training context may moderate adaptive outcomes; interventions implemented during competitive periods often coincide with accumulated fatigue and competing tactical demands, potentially blunting power-oriented adaptations (Malone et al., 2017). Well-trained athletes, in particular, may exhibit a ceiling effect whereby nonspecific stimuli such as common RST provide insufficient overload to further augment VJ performance (Seitz et al., 2014). Methodologically, variations in jump testing protocols and the absence of biomechanical data—such as rate of force development or lower limb stiffness—further complicate the interpretation of interventional effects (Cormie et al., 2011). Collectively, these findings suggest that linear RST lacks the specific neuromechanical stimuli required to enhance vertical jump performance. Future studies should examine whether integrating directional changes, resisted sprints, or plyometric components into RST regimens may elicit more meaningful improvements in vertical power expression (Alcaraz et al., 2018; Asadi et al., 2016).

### 4.4 Effects of field-based RST on CoD ability

This meta-analysis demonstrates that field-based RST induces moderate improvements in change of direction (CoD) performance among athletes (effect size g = −0.7). The enhancement in COD was significantly associated with optimized kinetic parameters (e.g., ground reaction impulse) and refined kinematic characteristics (e.g., step length and step frequency) (Sheppard and Young, 2006). Compared to linear sprinting, COD require athletes to execute rapid deceleration and reacceleration from initial speeds exceeding 20 km/h, thereby imposing greater physiological demands on eccentric braking capacity and concentric power output (Chelly and Denis, 2001).

From a biomechanical perspective, the beneficial effects of RST on COD ability primarily stem from synergistic neuromuscular and morphological adaptations: the neuromuscular system enhances motor unit recruitment efficiency, optimizes firing frequency, and promotes synchronized activation of fast-twitch fibers, collectively improving rate of force development and reactive strength (Ross and Leveritt, 2001; Creer et al., 2004). Concurrently, the transformation toward type IIa muscle fiber profiles and increased muscle cross-sectional area further augment force-generation capacity (Dawson et al., 1998). Notably, several incorporated RST protocols included directional change components whose deceleration-reacceleration patterns and force-vector characteristics closely matched those of COD tasks, potentially explaining the effective transfer of training effects.

Only one included study failed to demonstrate significant improvements, potentially due to the shorter sprint distance (18 m) insufficient to elicit adequate neuromuscular adaptation, combined with a linear training design lacking COD-specific biomechanical stimuli (Sanchez-Sanchez et al., 2019). Future research should systematically integrate multidirectional movement patterns (e.g., lateral cutting, diagonal acceleration) into RST protocol to enhance biomechanical alignment with target maneuvers, thereby more effectively developing braking control capacity and multidirectional acceleration performance.

### 4.5 Effects of field-based RST on aerobic capacity and high-intensity running performance

Field-based RST induces divergent adaptive responses in athletes: while it fails to elicit significant improvements in aerobic capacity (VO_2_max), it substantially enhances high-intensity running performance (g = 0.97), as consistently demonstrated in soccer-specific endurance assessments such as the Yo-Yo IR1/IR2 tests and the 30-15 Intermittent Fitness Test (30-15 IFT). In contrast to recent findings (Thurlow et al., 2024), the present meta-analysis did not identify a positive effect of RST on VO_2_max. This discrepancy in results may be attributed to several methodological and physiological factors. First, Sanchez-Sanchez et al. (2019) demonstrated only marginal improvements in VO_2_max among adolescent athletes (mean age 14.7 ± 0.5 years) following RST, which contrasts sharply with the more pronounced adaptations typically observed in adult populations. This suggests that maturational status may modulate training adaptations, as the developing cardiopulmonary system in youth athletes may constrain central cardiovascular plasticity. Second, methodological heterogeneity—including variations in VO_2_max assessment protocols (ranging from direct gas analysis to Yo-Yo IR1/IR2 –based estimations) and differences in RST programming parameters—likely contributes to the inconsistent outcomes across studies. Furthermore, from a physiological perspective, the absence of VO_2_max enhancement may be attributed to the inherent characteristics of RST protocols, which prioritize neuromuscular activation, phosphocreatine resynthesis, and anaerobic energy metabolism over central cardiovascular loading (Buchheit and Laursen, 2013a). Since VO_2_max is primarily determined by cardiac output and oxygen transport capacity, the characteristic short-duration sprints (≤6 s) coupled with incomplete recovery periods in RST provide insufficient stimulus for plasma volume expansion or stroke volume improvement—key determinants of VO_2_max (Gibala et al., 2012).

Notably, RST produces marked improvements in HIR performance, reflecting its efficacy in optimizing peripheral physiological adaptations crucial for intermittent sports. These adaptations include increased mitochondrial volume, enhanced calcium ion cycling efficiency (Tjønna et al., 2008), elevated oxidase activity (Iaia et al., 2017), and improved lactate clearance capacity, collectively promoting metabolic efficiency. Crucially, Yo-Yo IR1/IR2 and 30-15IFT tests evaluate not merely aerobic capacity but integrate anaerobic metabolism, neuromuscular coordination, and psychological tolerance—dimensions specifically targeted by RST. Krustrup et al. (2003) established that Yo-Yo IR Test performance correlates significantly with high-intensity activity during actual match play. Match analyses confirm soccer players frequently execute repeated sprints with directional changes (Bravo et al., 2008), and RST’s incorporation of acceleration, deceleration, and CoD elements closely mimics these match-specific biomechanical patterns, aligning with the principle of task specificity. Thus, although RST does not enhance VO_2_max as an indicator of aerobic power, it systematically improves the physiological determinants of high-intensity intermittent performance, validating its application for athletes reliant on repeated explosive efforts rather than pure aerobic capacity.

## 5 Limitations

Several limitations of this systematic review and meta-analysis should be considered when interpreting the findings. First, due to the limited number of included studies, it was not feasible to conduct subgroup analyses based on potential influencing factors such as sex, age, maturity status, and competitive level (e.g., elite vs. sub-elite). This constraint hinders our ability to thoroughly explore the differential effects of various RST protocols and to refine application strategies accordingly. Second, some studies did not specify the training phase (e.g., preparatory or competitive period) during which the RST intervention was implemented, which may affect the accurate interpretation of training adaptation and recovery outcomes.

## 6 Future perspectives

Future research should aim to precisely elucidate the dose-response relationships of RST, systematically investigating the differential effects of various training variable combinations—such as sprint distance/duration, inter-set rest periods, work-to-rest ratios, number of repetitions, and training frequency—on athletes of different levels, including elite and youth populations. Long-term longitudinal studies are warranted to uncover the sustained mechanisms underlying RST-induced physiological adaptations and enhancements in athletic performance.A key focus should be placed on examining how individual factors—such as age, sex, maturity status, and competitive level—moderate training adaptations. Particular attention is needed regarding the constraints imposed by the ongoing development of the cardiopulmonary system in adolescent athletes on cardiovascular responses to high-intensity training, informing the design of RST protocols appropriate for their developmental stage. From a training content perspective, incorporating more sport-specific movements—including changes of direction, deceleration, and re-acceleration—can enhance the ecological validity of RST relative to the actual demands of soccer match play. Concurrently, exploring the integration of RST with strength and power training modalities, such as resisted sprint training, may improve acceleration performance. Further investigation is needed into the mechanisms for coordinating concurrent power and endurance training during the competitive period.Methodologically, standardized physiological assessment protocols—such as uniform direct measurement of VO_2_max—should be adopted. Additionally, there is a need to develop biomarkers capable of sensitively reflecting microscopic adaptations to RST, including mitochondrial function, calcium ion cycling, oxidase activity, and lactate clearance capacity. This will help further elucidate the underlying neuromuscular and metabolic mechanisms.Furthermore, future studies should clearly define the training cycle context—such as preparatory versus competitive phases—in which RST is implemented, and examine how these phases influence training adaptations and recovery outcomes. Strengthening empirical research within authentic training environments is essential to strike an optimal balance between maintaining sprint velocity and managing fatigue, thereby refining RST programming to develop precise and periodized training plans for athletes across different tiers.

## 7 Practical implications

This review confirms that field-based RST is an effective tool for improving specific physical performance qualities in soccer players. Coaches and strength and conditioning professionals can apply RST to reliably enhance athletes' short-distance sprint performance, RSA, CoD ability, and high-intensity running endurance. For training objectives aimed at improving VJ height or VO_2_max, other more targeted training methods (such as plyometrics or long-interval training) should be prioritized.To better transfer gains to the agility demands of soccer match-play, sport-specific movement patterns such as directional changes (e.g., 45° or 90° cuts), rapid decelerations, and re-accelerations can be incorporated into basic linear sprint patterns. This adjustment enhances the ecological validity of the training relative to match scenarios. For well-trained athletes, if the stimulus from conventional RST becomes insufficient, progression can be considered by adding resistance (e.g., using parachutes or sleds) or integrating complex reactive tasks. The findings of this study highlight a significant knowledge gap: a clear dose-response relationship has not been established. In practice, this means that coaches must still rely on experience and individual athlete monitoring when adjusting key variables such as sprint distance, rest intervals, and the number of sets and repetitions. Future research should focus on clarifying the specific relationships between these variables and different performance outcomes to develop more optimized and individualized prescriptions.

## 8 Conclusion

Based on the findings of this systematic review and multilevel meta-analysis, field-based RSTdemonstrates significant efficacy in enhancing key physical performance qualities in soccer players, particularly short-sprint ability, RSA, CoD ability and high-intensity running performance. The improvements in these domains are likely mediated by RST-induced neuromuscular adaptations, metabolic enhancements, and its high degree of task specificity to the sprinting demands of soccer. In contrast, RST appears to have limited and inconsistent effects on VJ performance, and aerobic capacity. To optimize the application of RST, future research should prioritize elucidating precise dose-response relationships, accounting for individual differences such as age, sex, and maturation status, and designing training protocols that incorporate sport-specific movements (e.g., deceleration, re-acceleration, and directional changes). Furthermore, standardizing physiological assessment methods and investigating the long-term adaptations to RST across different training periods will be crucial for translating evidence into targeted, effective training prescriptions in soccer.

## Data Availability

The original contributions presented in the study are included in the article/Supplementary Material, further inquiries can be directed to the corresponding authors.
