# Supplementary material for: The effects of field-based repeatedsprint training on physical performance in soccer players: a systematic review and multilevel meta-analysis
Source: Front Physiol. 2026 Mar 30;17:1745959. doi: 10.3389/fphys.2026.1745959 (PMC13070835; doi:10.3389/fphys.2026.1745959)
Supplement: Supplementary file 1 [file DataSheet1.docx]

**Journal:** Frontiers in Physiology

**Title:**The Effects of Field-Based Repeated-Sprint Training on Physical Performance in Soccer Players: A Systematic Review and Multilevel Meta-Analysis

**Authors:** Anqi Chen^1,2†^ , Mingnan Zhuang^3,4†^ , Zijing Huang^5^, Liang Zhao^2,6*^, Leibo Wang^7*^

**Affiliations:** ^1^Graduate School of Shandong Sport University, Jinan, China;

^2^Digital Physical Training Laboratory, Shandong Sport University, Rizhao, China;

^3^School of Sport Training, Tianjin University of Sport, Tianjin, China;

^4^School of Human Movement Science, Hebei Sport University, Shijiazhuang, China;

^5^School of Physical Performance, Shanghai University of Sport;

^6^School of Competitive Sports, Shandong Sport University, Rizhao, China;

^7^National Football Academy, Shandong Sport University, Rizhao, China;

*The author are co-corresponding author

^†^ Anqi Chen and Mingnan Zhuang have made equal contributions.

**Supplement 1. Search strategy**

Table 1 Number of hits for the complete search strategy in the databases.

| Database | Complete Search Strategy | Hits August 2025 |
| --- | --- | --- |
| Web of Science | (AB=(RST OR "repeat* sprint*" OR "intermittent sprint*" OR "multiple sprint*" OR "sprint interval training") AND ("exercise" OR "ability" OR "training") AND ("team sport" OR "soccer") AND ("physiological" OR "metabolic" OR "fatigue" OR "performance") NOT ("treadmill" OR "cycling" OR "swimming")) | 888 |
| PubMed | ("RST" [Title/Abstract] OR "repeat* sprint*" [Title/Abstract] OR "intermittent sprint*" [Title/Abstract] OR "multiple sprint*" [Title/Abstract] OR "sprint interval training" [Title/Abstract]) AND ("exercise" [Title/Abstract] OR "ability" [Title/Abstract] OR "training" [Title/Abstract]) AND ("team sport" [Title/Abstract] OR "soccer" [Title/Abstract]) AND ("physiological" [Title/Abstract] OR ("metabolic" [Title/Abstract] OR "fatigue" [MeSH Terms] OR "performance" [Title/Abstract])) NOT ("treadmill"[Title/Abstract] OR "cycling"[Title/Abstract] OR "swimming"[Title/Abstract]) | 697 |
| Scopus | (ABS(exercise) OR (ability) OR (training) ) AND (ABS(team sport) OR (soccer)) AND (ABS(physiological) OR (metabolic) OR (fatigue) OR (performance)) NOT (ABS(treadmill) OR (cycling) OR (swimming)) | 212 |
| EBSCOhost databases | AB(RST OR "repeat* sprint*" OR "intermittent sprint*" OR "multiple sprint*" OR "sprint interval training") AND ("exercise" OR "ability" OR "training") AND ("team sport" OR "soccer") AND ("physiological" OR "metabolic" OR "fatigue" OR "performance") NOT ("treadmill" OR "cycling" OR "swimming") | 1081 |

**Supplement 2. The forest plots of meta-analysis**

**2.1 Forest plots of assessing the effects of field-based on short-sprint ability**

Figure S1. The analysis for the effect of RST on short-sprint ability

**
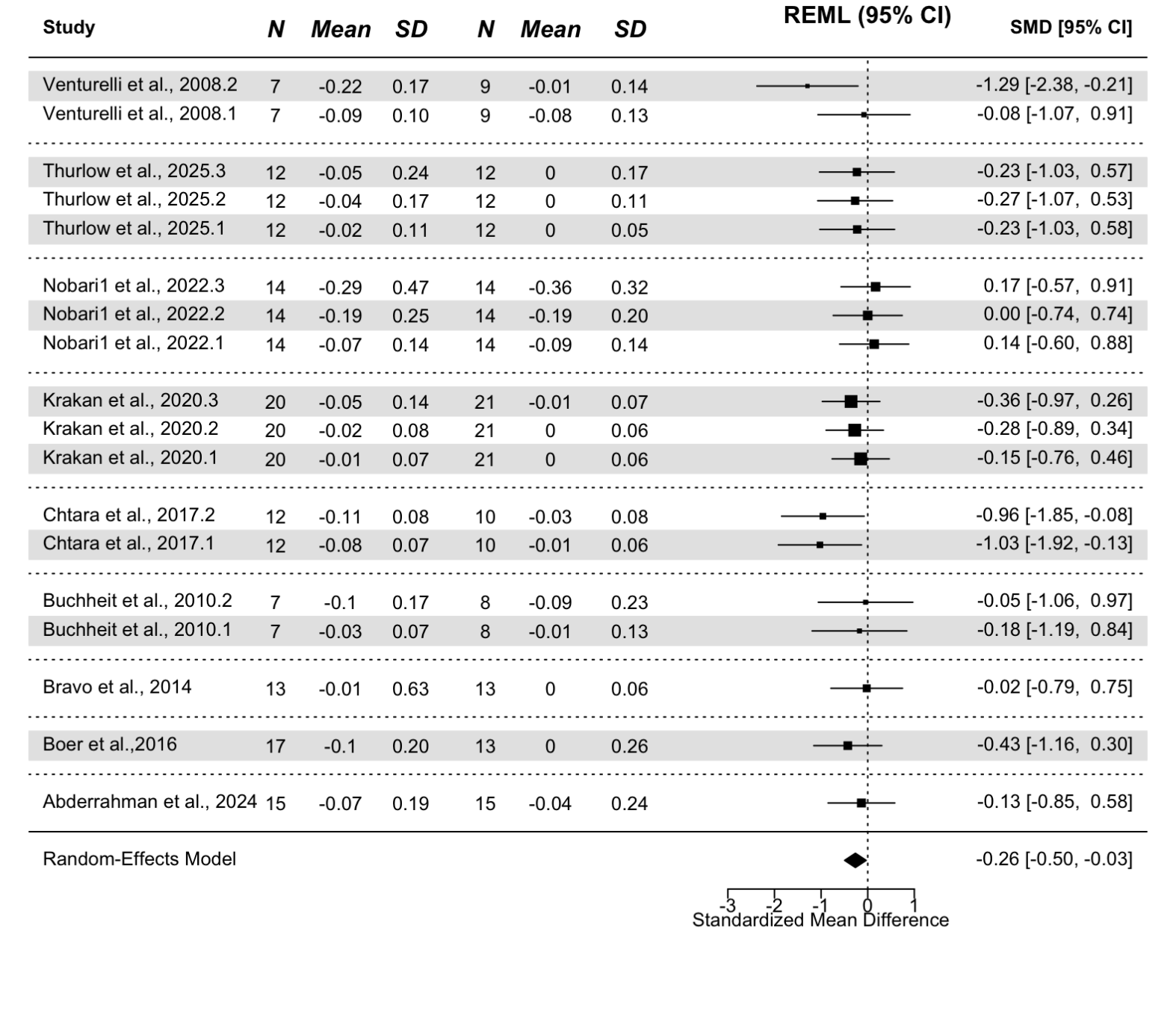
**

**2.2 Forest plots of assessing the effects of field-based on Repeated Sprint Ability(RSA)**

Figure S2. The analysis for the effect of RST on RSA

**
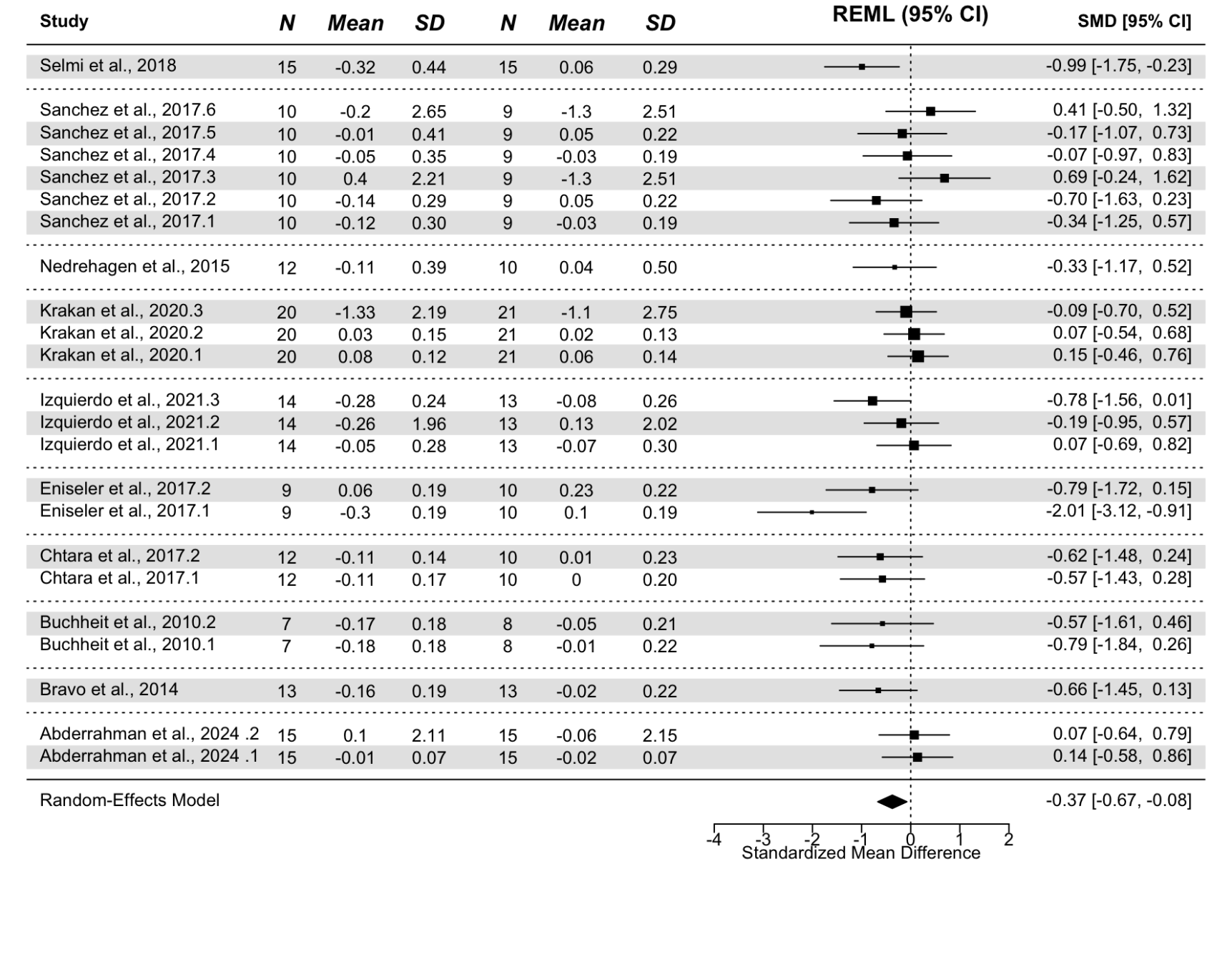
**

**2.3Forest plots of assessing the effects of field-based vertical jump performance**

Figure S3. The analysis for the effect of RST on vertical jump performance


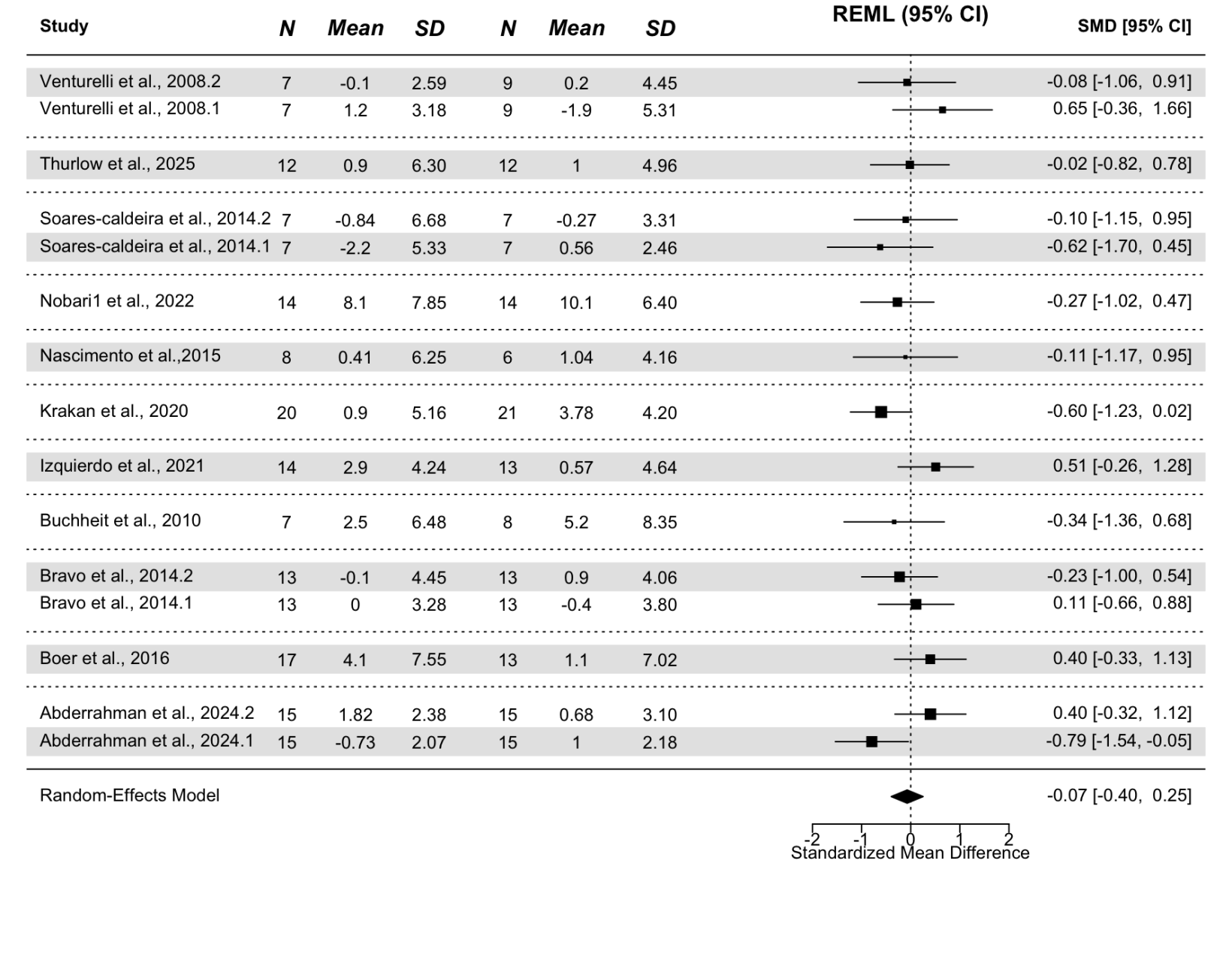


**2.4Forest plots of assessing the effects of field-based onchange of direction (CoD) ability**

Figure S4. The analysis for the effect of RST on CoD


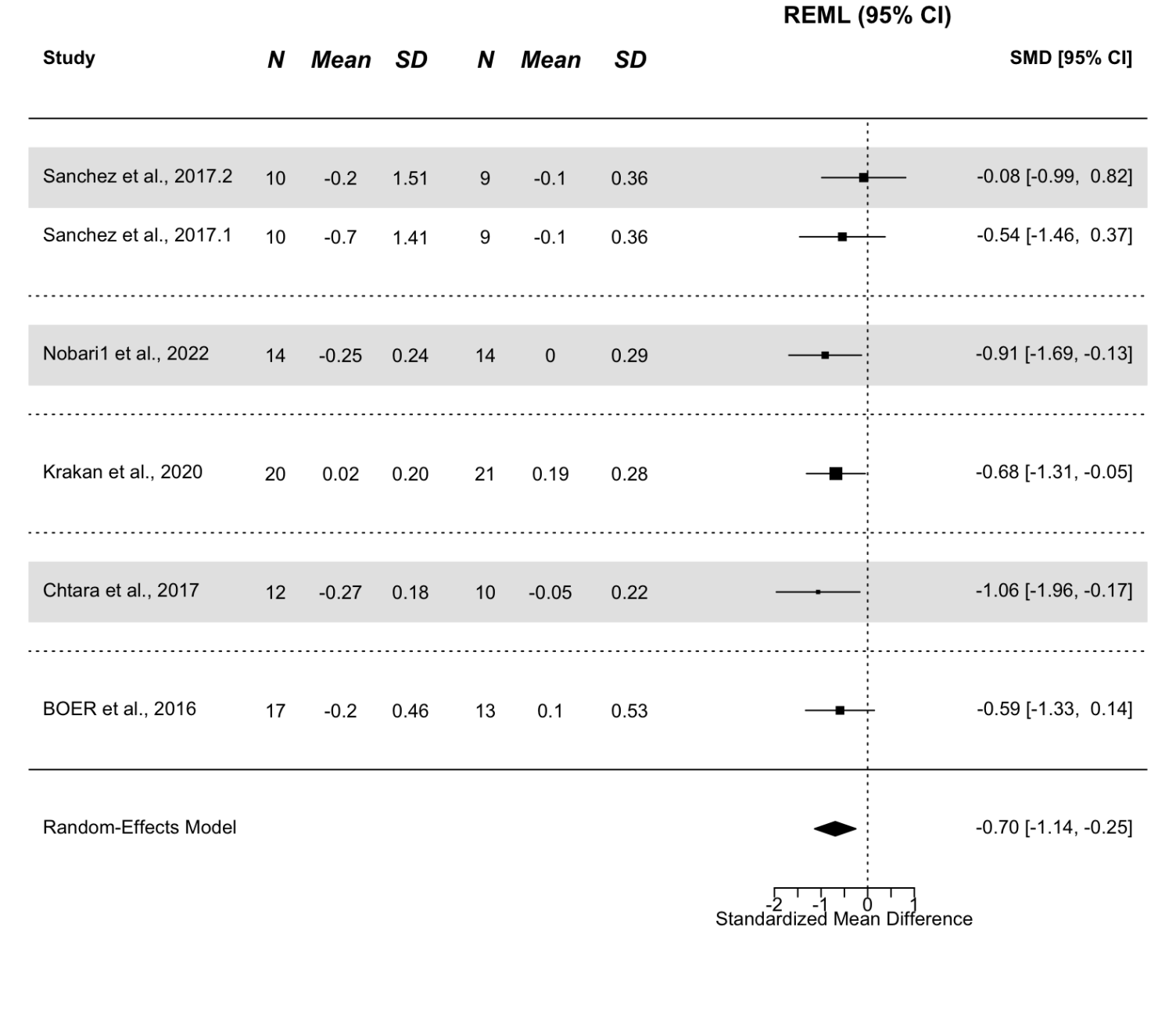


**2.5Forest plots of assessing the effects of field-based on aerobic capacity**

Figure S5. The analysis for the effect of RST on aerobic capacity


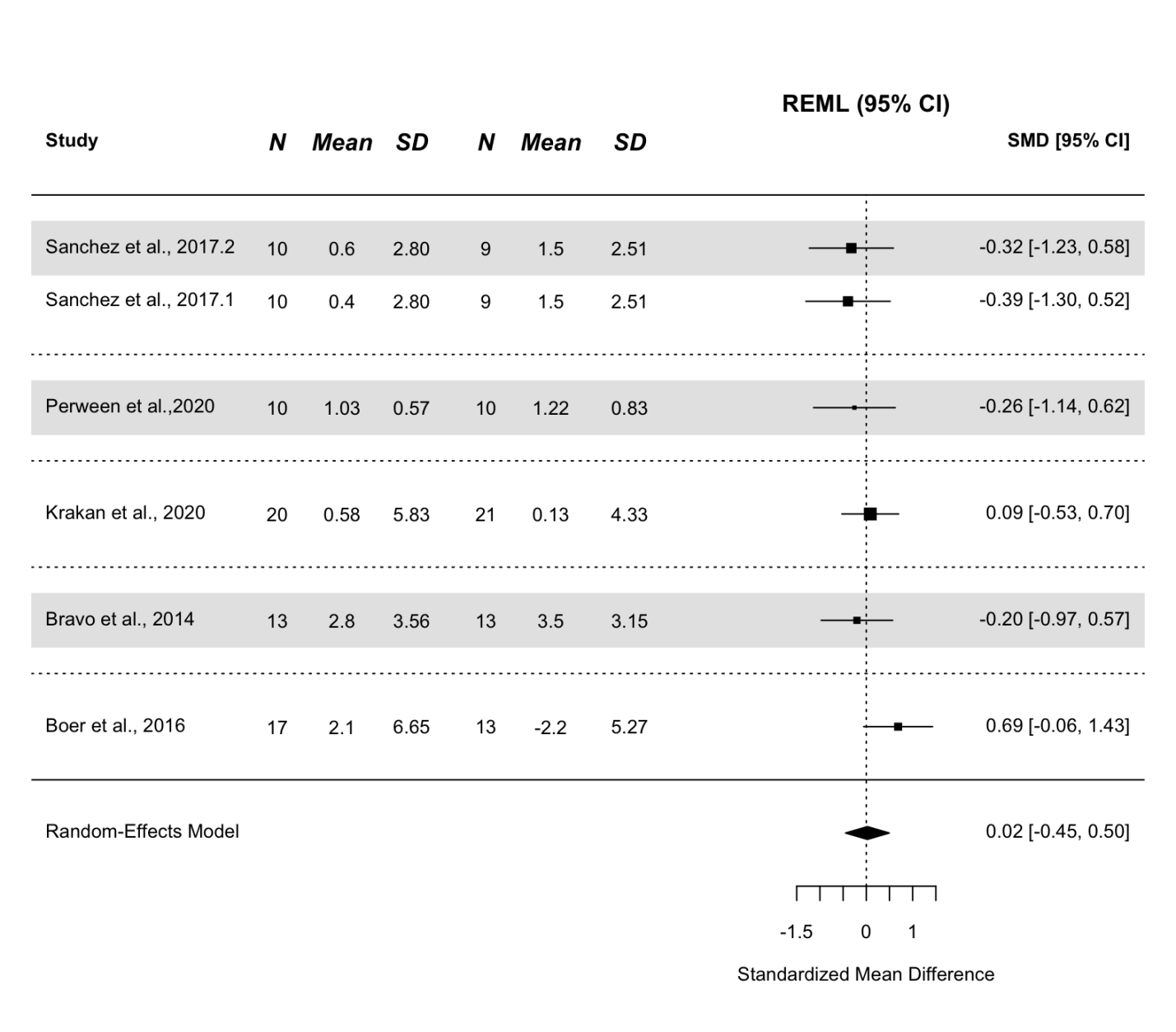


**2.6Forest plots of assessing the effects of field-based on high intensity running (HIR) performance**

Figure S6. The analysis for the effect of RST on HIR


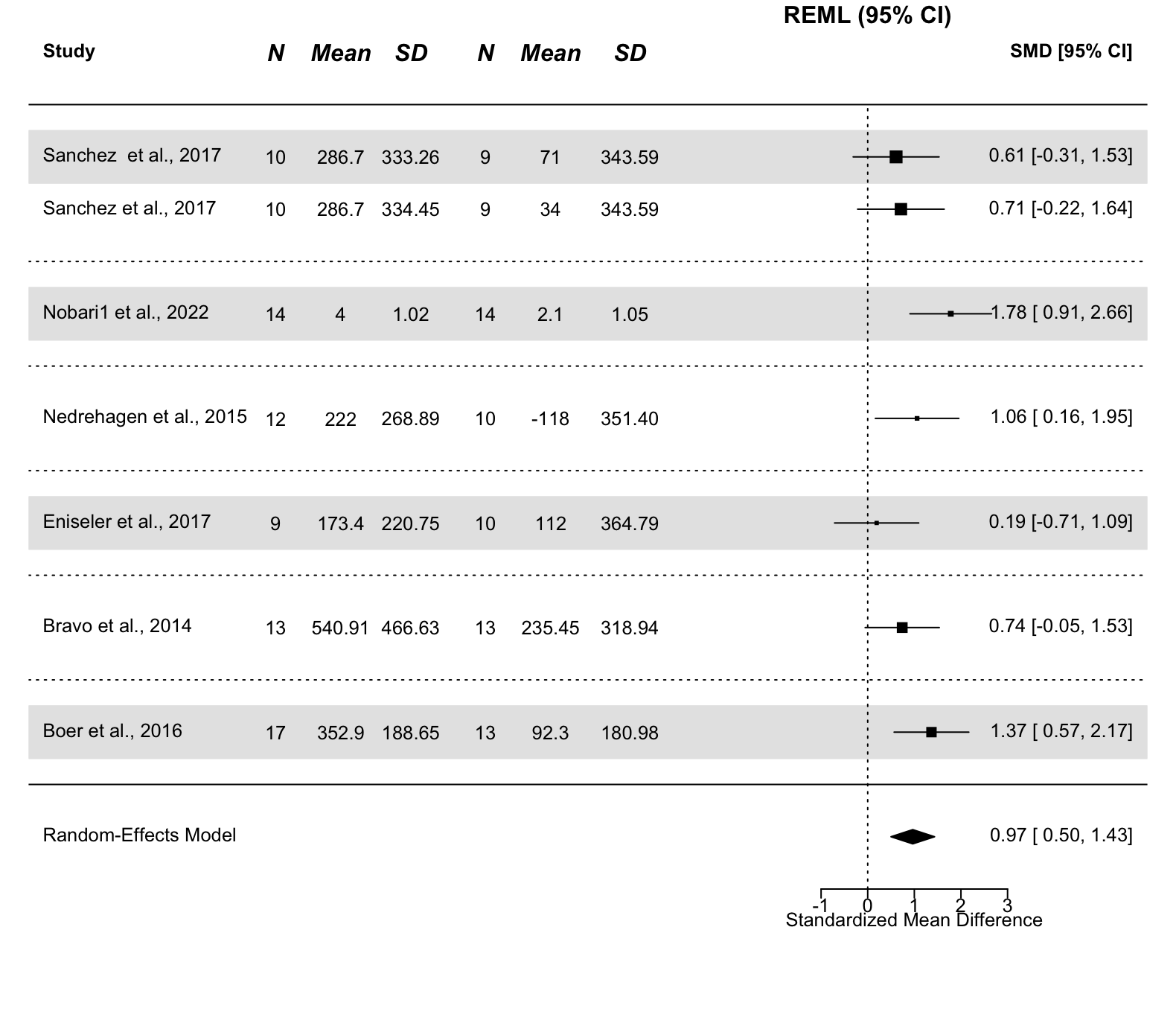


**Supplement 3. GRADE Evidence Summary**

Table S1. GRADE Summary of Evidence for RST vs CON

| Outcome | Participants (RCTs) | **GRADE Evidence Certainty Assessment** | | | | | Hedge’s *g* (95% CI) | Certainty* |
| --- | --- | --- | --- | --- | --- | --- | --- | --- |
|  |  | Risk of bias | Inconsistency | Indirectness | Imprecision | Other concerns |  |  |
| **Aerobic Exercise vs No-Training Control Group** | | | | | | | | |
| **Short-Sprint Ability** | **491**  **(9 RCT)** | **Not serious** | **Not serious** | **Not serious** | **Not serious** | **None** | **-0.26 (-0.50 to -0.03)** | ⨁⨁◯◯ **Moderate** |
| **RSA** | **568**  **(9 RCT)** | **Not serious** | **Serious** | **Not serious** | **Not serious** | **None** | **-0.37 (-0.67 to -0.08)** | ⨁⨁◯◯ **Moderate** |
| **VJ** | **351**  **(11 RCT)** | **Not serious** | **Not serious** | **Not serious** | **Not serious** | **None** | **-0.07(-0.40 to -0.25)** | ⨁⨁⨁◯ **Moderate** |
| **CoD** | **159**  **(5 RCT)** | **Not serious** | **Not serious** | **Not serious** | **Not serious** | **None** | **-0.70（-1.14 to -0.25）** | ⨁◯◯◯ **Moderate** |
| **Aerobic Capacity** | **155**  **(9 RCT)** | **Not serious** | **Not serious** | **Not serious** | **Not serious** | **None** | **-0.02（-0.45 to -0.50）** | ⨁⨁◯◯ **High** |
| **HIR** | **163**  **(6 RCT)** | **Not serious** | **Serious** | **Not serious** | **Not serious** | **None** | **0.97（0.50 to 1.43）** | ⨁⨁◯◯ **Moderate** |
| **Abbreviations: RSA=repeated sprint ability; VJ=vertical jump；CoD=change of direction；HIR=high intensity running**  † **GRADE levels of certainty**  High: We are very confident that the true effect lies close to the estimate.  Moderate: The true effect is likely close to the estimate, but there is a possibility it is substantially different.  Low: Our confidence in the effect estimate is limited—the true effect may be substantially different.  Very low: We have very little confidence in the effect estimate. | | | | | | | | |
